# RMzyme: regulations of RNA-modifying enzymes in humans

**DOI:** 10.1038/s41392-025-02568-2

**Published:** 2026-02-12

**Authors:** Ruihan Luo, Haixia Xu, Qingbo Zhou, Shanli Ding, Min Qiang, Jianguo Wen, Pora Kim, Xiaojuan Yang, Yunshi Cai, Kunlin Xie, Jiang Zhu, Yungang Xu, Tian Lan, Xiaobo Zhou, Hong Wu

**Affiliations:** 1 Laboratory of Hepatic AI Translation, Frontier Science Center for Disease-Related Molecular Network, West China Hospital, Sichuan University, Chengdu, China; 2 Center for Computational Systems Medicine, McWilliams School of Biomedical Informatics, The University of Texas Health Science Center at Houston, Houston, TX USA; 3 West China Biomedical Big Data Center, West China Hospital, Sichuan University, Chengdu, China; 4 Graduate School of Biomedical Sciences, The University of MD Anderson Cancer Center, Houston, TX USA; 5 Department of Cell Biology and Genetics, School of Basic Medical Sciences, Xi’an Jiaotong University Health Science Center, Xi’an, China; 6 Department of General Surgery, West China Hospital, Sichuan University, Chengdu, China; 7 Liver Transplant Center, Transplant Center, West China Hospital, Sichuan University, Chengdu, China; 8 McGovern Medical School, The University of Texas Health Science Center at Houston, Houston, TX USA

**Keywords:** Molecular medicine, Genome informatics

## Abstract

RNA modifications represent a dynamic layer of gene expression regulation, RNA stability, and translation with profound implications for cellular function and disease. However, the critical regulation and functions of RNA-modifying proteins (RMPs) remain poorly understood. Here, we present a large-scale characterization of RMPs through 378 multiomics datasets encompassing genomics, bulk and single-cell transcriptomics, epitranscriptomics, proteomics, and posttranslational modifications (PTMs) across 63 human tissues. Our analysis of experimental perturbations of RMPs revealed dynamic differential modification peaks and expressed genes. We applied nonnegative matrix factorization to annotate RMP-mediated cell types in single-cell transcriptomes. Functional annotations in acute myeloid leukemia (AML) revealed RMPs such as ALKBH5 as critical mediators of m6A dynamics, influencing pathways involved in translation initiation, immune regulation, and tumorigenesis. We revealed cell type-specific modification patterns, including those in ALKBH5-enriched AML stem cells with special ligand‒receptor interactions and genetic variations modulated by m6A. We integrated proteogenomic data to uncover PTM-associated regulatory, mutation, and protein‒protein interaction networks linked to RMPs. We developed RMzyme, a platform that consolidates our findings and provides insights into RMPs and their downstream effects. This resource is expected to facilitate biomedical research into the molecular mechanisms of human diseases through the lens of RNA modifications and multiomics data integration.

## Introduction

Most RNA modifications are reversible chemical changes introduced to RNA molecules following their synthesis, which regulate RNA metabolism and fate and affect many cellular and biological processes.^1^ To date, over 170 chemical modifications in RNA have been identified,^2^ constituting the complexity of the epitranscriptome. Numerous studies focusing on the epitranscriptome regulated by RNA modification have demonstrated that related regulators are involved in various human diseases, including nonalcoholic fatty liver disease, azoospermia, and heart failure, especially in human cancers.^3^ Nevertheless, the critical regulation and functions of RNA-modifying proteins (RMPs) remain poorly understood.

**Fig. 1:**
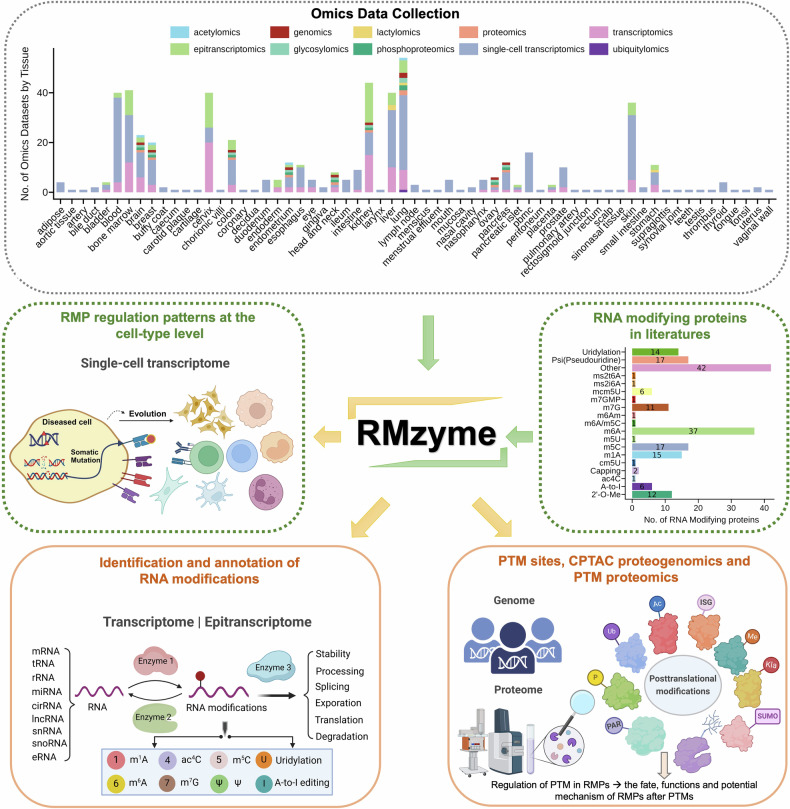
Overview of the RMzyme database. This platform includes multiomics datasets collected from 63 human tissues. The RMzyme knowledgebase features three search modules centered ~179 human RNA-modifying proteins (RMPs), offering detailed information concerning the posttranscriptional modification machinery at the cell-type level, the regulators and downstream targeting genes, and the protein and posttranslational modification (PTM) at the site and protein levels. Schematics created with BioRender.com

Currently, there are several available databases regarding RNA modifications, such as directRMDB,^4^ m6A-atlas,^5^ m5C-atlas,^6^ m7GHub^7^ and RMBase,^2^ which focus on providing many high-confidence modification sites, and MODOMICS,^8^ which uses literature retrieval for ref. ^2^ validated information about modified residues. However, comprehensive characterization of the mechanisms that mediate the function of RNA modification enzymes in various physiological and pathological situations in humans is lacking. Additionally, proteins undergo chemical modifications termed posttranslational modifications (PTMs), which occur on amino acid side chains, as well as on terminal amino and carboxyl groups.^9^ PTMs affect protein structure, stability, activity, localization, or solubility.^10^ Hence, knowledge of PTMs that can control the biology of these RNA modification regulators is important but still limited. To this end, we constructed the RMzyme, a knowledge base concerning the regulation of RMPs in humans, to provide a comprehensive resource for understanding the role of RMPs in the regulation of biological functions and human disease progression (Fig. 1). Our platform (https://rh-luo.cn/RMzyme) illustrates different layers of 179 human RMPs from 18 major RNA modifications (RMs) that have been reported in publications, facilitating a deep understanding of sophisticated regulatory networks and aiding in the development of small-molecule inhibitors that target specific RMPs and the identification of novel drug targets.

To accomplish this goal, we annotated and analyzed 382 multiomics datasets, including genomics, bulk and single-cell transcriptomics, epitranscriptomics, proteomics, acetylomics, phosphoproteomics, glycoproteomics, lactylomics and ubiquitylomics datasets, encompassing 63 human tissue types through PubMed queries and obtained from multiple data repositories (Fig. 1). Our analysis of omics data involved rigorous preprocessing and quality control steps. First, we utilized the nonnegative matrix factorization (NMF) algorithm to characterize the effect of RMP regulator expression on 16 major cell types from 264 human single-cell RNA-sequencing (scRNA-seq) datasets. We annotated and visualized NMF RNA modification-mediated subtypes on the basis of 179 regulatory enzymes of RMs. We then identified differentially expressed genes (DEGs) by comparing the diseased and healthy groups for the main cell subtypes as well as RNA modification-related DEGs among the NMF subtypes. To understand RNA modification heterogeneity in single cells and the distinct RNA modification-mediated patterns that contribute to disease progression, we performed functional enrichment analysis of KEGG pathways for each NMF RNA modification-related cluster, followed by intercellular communication and single-cell somatic mutation analyses. Second, to reveal the effects of regulatory enzymes on human RMs and identify the key downstream targets of RNA modification mediators, we analyzed 106 bulk transcriptome and epitranscriptiome datasets from 62 human cell lines with or without knockout, knockdown, overexpression, mutation, inhibition and rescue of RMPs. We identified differential RNA modification peaks and DEGs between different sample conditions and annotated differential binding peaks. We further provided functional annotations for the identified transcripts on the basis of signatures from the MsigDB database. We also present potential RNA-binding protein (RBP)-binding events associated with RNA modification sites. Additionally, drug information targeting RNA regulatory enzymes and modified RNA is provided in RMzyme. Third, we present information on PTMs on RMPs from the literature reviews and PTM-related databases. Using proteogenomic data across 11 cancer types from the Clinical Proteomic Tumor Analysis Consortium (CPTAC), we identified protein‒protein interactions with RNA-modifying regulators on the basis of their correlation analyses of protein abundance, phosphorylation, acetylation, ubiquitination and glycosylation levels. Additionally, we analyzed the mutation patterns of RMPs on the basis of the CPTAC DNA-seq data. We performed differential expression analyses of RNA, protein and PTM levels in CPTAC samples compared with normal samples.

To demonstrate the practical utility of the RMzyme, we focused on acute myeloid leukemia (AML), a hematologic malignancy known to be regulated by RMs.^11,12^ In this application, we systematically integrated bulk and single-cell transcriptomic and epitranscriptomic datasets from AML studies. This enabled us to (i) validate previously reported RNA modification regulators, such as METTL3 and ALKBH5, along with their associated downstream pathways and (ii) identify novel RNA modification-mediated cell subpopulations, including ALKBH5+ stem cells, as well as potential novel biomarkers and therapeutic targets, such as ZNF503. Our AML-focused analysis highlights RMzyme’s ability to recapitulate established biological findings and generate new hypotheses for future mechanistic studies. As explained here, RMzyme offers comprehensive reference information on human RNA-modifying proteins and provides a platform for mapping RNA modification-mediated regulation across diverse tissues and diseases. This resource not only supports large-scale omics data integration but also enables disease-specific investigations. Owing to the broad interest from the research community, RMzyme is highly expected to be a valuable repository facilitating biomedical studies focusing on the regulatory mode of human diseases and its underlying mechanisms via omics data.

## Results

### Characterization of the RNA modification map across cell lines

Our motivations in this work are to understand how RMs decode changes in the macro/microenvironment to initiate proper cellular responses and how RNA-modifying enzymes are involved in the regulation of physiology and human diseases. Hence, by using epitranscriptomic datasets derived from RNA immunoprecipitation-based sequencing methods (e.g., MeRIP-seq and m6A-seq), we first identified transcriptome-wide RNA modification sites and peaks across different cell types. After sequencing reads were aligned to the reference genome, we called peaks in transcript coverage in the immunoprecipitation (IP) fraction relative to the input control, resulting in over 2 million sites for 8 human RNA modification types across 122 different conditions in 42 cell lines. They included 9038 sites for ac4C, 18,129 sites for m1A, 190,847 sites for m5C, 41,083 sites for m5U, 2,546,308 sites for m6A, 146,906 sites for m6Am, 16,544 sites for m7G and 4,011 sites for pseudouridine (Table 1). Across all epitranscriptomic datasets, we identified 4011 ~ 1,813,239 unique RNA modification sites (defined by genomic coordinates and strands) spanning 8 RNA modification types (Table 2). Among these, m6A was the most abundant, followed by m5C and m6Am. To account for dataset imbalance among modification types, we normalized the number of unique modification sites by the number of conditions analyzed per type. We observed striking variability in the number of unique RNA modification sites across modification types and datasets (Table 2). Normalization to the number of experimental contrasts revealed that m5U modifications presented the highest density per contrast, followed by m6Am and m6A modifications, suggesting highly focused deposition or detection. The high normalized count of m6A still indicates its widespread presence across the transcriptome, aligns with its known regulatory roles in RNA splicing, stability, translation, etc. We also note that differences in the number of identified modification sites across types partly reflect variation in detection sensitivity and methodological maturity among profiling techniques. Hence, our data summary does not imply absolute biological dominance of m⁶A over other modifications but represents a combination of true biological prevalence and method-driven detection bias.

**Table 1:** Statistical analysis of RNA modification sites across 42 cell lines via RMzyme

Cell line	ac4C	m1A	m5C	m5U	m6A	m6Am	m7G	Psi	Sum
A375	n.d.	n.d.	n.d.	n.d.	14,543	n.d.	n.d.	n.d.	14,543
A549	n.d.	n.d.	3250	n.d.	141,240	n.d.	3527	n.d.	148,017
AGS	n.d.	n.d.	n.d.	n.d.	6018	n.d.	n.d.	n.d.	6018
BGC823	n.d.	n.d.	n.d.	n.d.	612	n.d.	n.d.	n.d.	612
Caco-2	n.d.	n.d.	n.d.	n.d.	4128	n.d.	n.d.	n.d.	4128
EndoC-bH1	n.d.	n.d.	n.d.	n.d.	29,402	n.d.	n.d.	n.d.	29,402
Fibroblasts	n.d.	n.d.	n.d.	n.d.	12,475	n.d.	n.d.	n.d.	12,475
GSC11	n.d.	n.d.	n.d.	n.d.	30,792	n.d.	n.d.	n.d.	30,792
H322	n.d.	n.d.	n.d.	n.d.	495	n.d.	n.d.	n.d.	495
H9	n.d.	n.d.	16,360	n.d.	n.d.	n.d.	n.d.	n.d.	16360
HaCaT	n.d.	n.d.	n.d.	n.d.	56,554	n.d.	n.d.	n.d.	56,554
HCCLM3	n.d.	n.d.	n.d.	n.d.	2289	n.d.	n.d.	n.d.	2289
HCT116	n.d.	n.d.	n.d.	n.d.	45,703	n.d.	n.d.	n.d.	45,703
HCT15	n.d.	n.d.	15,106	n.d.	n.d.	n.d.	n.d.	n.d.	15,106
HEC-1-A	n.d.	n.d.	n.d.	n.d.	45,291	n.d.	n.d.	n.d.	45,291
HEK293	n.d.	n.d.	138,952	41,083	32,902	n.d.	n.d.	661	213,598
HEK293A-TOA	n.d.	n.d.	n.d.	n.d.	20,724	n.d.	n.d.	n.d.	20,724
HEK293T	n.d.	10,783	295	n.d.	511,872	93,107	n.d.	n.d.	616,057
HeLa	9038	7346	16,468	n.d.	408,058	n.d.	6563	3350	450,823
HepG2	n.d.	n.d.	n.d.	n.d.	224,838	n.d.	6454	n.d.	231,292
hESCs	n.d.	n.d.	n.d.	n.d.	203,983	n.d.	n.d.	n.d.	203,983
HG3	n.d.	n.d.	n.d.	n.d.	44,419	n.d.	n.d.	n.d.	44,419
HK2	n.d.	n.d.	n.d.	n.d.	62,177	n.d.	n.d.	n.d.	62,177
HTR8	n.d.	n.d.	n.d.	n.d.	12,288	n.d.	n.d.	n.d.	12,288
HUVECs	n.d.	n.d.	n.d.	n.d.	56,846	n.d.	n.d.	n.d.	56,846
IMR9n.d.	n.d.	n.d.	n.d.	n.d.	39,866	n.d.	n.d.	n.d.	39,866
K18n.d.	n.d.	n.d.	n.d.	n.d.	1655	n.d.	n.d.	n.d.	1655
MA9	n.d.	n.d.	n.d.	n.d.	150,540	n.d.	n.d.	n.d.	150,540
MDA-MB-231	n.d.	n.d.	n.d.	n.d.	22,621	n.d.	n.d.	n.d.	22,621
MEL624	n.d.	n.d.	n.d.	n.d.	n.d.	53,799	n.d.	n.d.	53,799
MM6	n.d.	n.d.	n.d.	n.d.	19,729	n.d.	n.d.	n.d.	19,729
MOLM13	n.d.	n.d.	n.d.	n.d.	10,086	n.d.	n.d.	n.d.	10,086
MONO-MAC-6	n.d.	n.d.	n.d.	n.d.	56,118	n.d.	n.d.	n.d.	56,118
NB4	n.d.	n.d.	n.d.	n.d.	80,207	n.d.	n.d.	n.d.	80,207
NOMO1	n.d.	n.d.	n.d.	n.d.	31,287	n.d.	n.d.	n.d.	31,287
PBT003	n.d.	n.d.	n.d.	n.d.	16,363	n.d.	n.d.	n.d.	16,363
PC-3	n.d.	n.d.	n.d.	n.d.	3700	n.d.	n.d.	n.d.	3700
SK-BR-3	n.d.	n.d.	n.d.	n.d.	1806	n.d.	n.d.	n.d.	1806
SU-DHL-8	n.d.	n.d.	n.d.	n.d.	11,743	n.d.	n.d.	n.d.	11,743
T24	n.d.	n.d.	416	n.d.	n.d.	n.d.	n.d.	n.d.	416
THP1	n.d.	n.d.	n.d.	n.d.	97,652	n.d.	n.d.	n.d.	97,652
UM-SCC-1	n.d.	n.d.	n.d.	n.d.	35,286	n.d.	n.d.	n.d.	35,286
Sum	9038	18,129	190,847	41,083	2,546,308	146,906	16,544	4011	2,972,866

“n.d.” stands for not determined and no publicly available data. More “n.d.” in each row (cell line) reflects the current research imbalance in the field, where m⁶A and a few well-studied modifications dominate the available datasets, whereas others (e.g., m⁵C, ψ, and ac⁴C) remain underexplored

**Table 2:** Unique RNA modification sites across the transcriptome in RMzyme

Modification type	Unique site count	Num of dataset	Normalized site count
m6A	1,813,239	92	19,709
m5C	175,678	22	7985
m6Am	141,564	4	35,391
m5U	41,083	1	41,083
m1A	18,129	3	6043
m7G	16,534	3	5511
ac4C	9038	1	9038
Psi	4011	2	2006

**Fig. 2:**
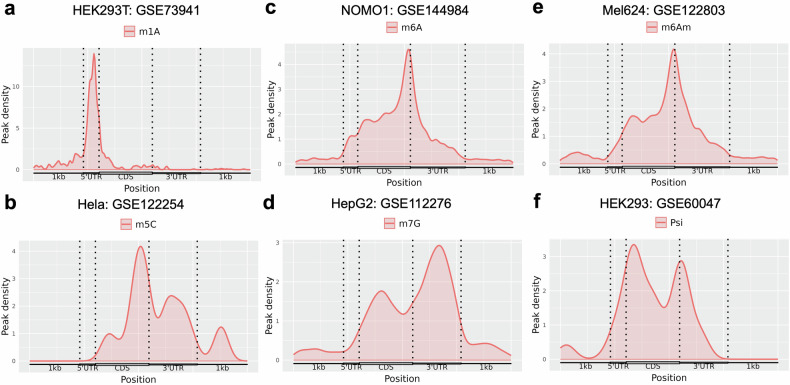
Metagene profiles of the distributions of m1A (**a**), m5C (**b**), m6A (**c**), m7G (**d**), m6Am (**e**) and pseudouridine (**f**) across mRNA segments in cell lines

Next, we described the distribution of RMP binding sites across the RNA segments, revealing a wide range of preferences for different RMs in the transcriptome. For example, the metagene profiles revealed that m1A binding sites were predominantly localized at 5′ untranslated regions (UTRs) and near the start codon. m5C tends to show enriched peaks in the CDS and 3′ UTR of mRNAs as well as enrichment at the start and stop codons and in noncoding RNA (ncRNA). Notably, m6A peaks were located around the start and stop codons within the 5′ UTR, 3′ UTR, CDS regions, and ncRNA. m7G is also widely distributed in the interior of mRNAs and ncRNAs. Pseudouridine is present primarily around the start and stop codons, within the CDS and 3′ UTR of mRNAs, and in ncRNAs (Fig. 2a–f, Supplementary Fig. 1a–f). Our analysis revealed substantial overlap and consistent positional enrichment with the original studies (Supplementary Fig. 1g). These results indicate that the distinct patterns of different RMs may closely correlate with their diverse biogenesis mechanisms.

### Detection and annotation of RNA modification peak changes among different conditions

**Fig. 3:**
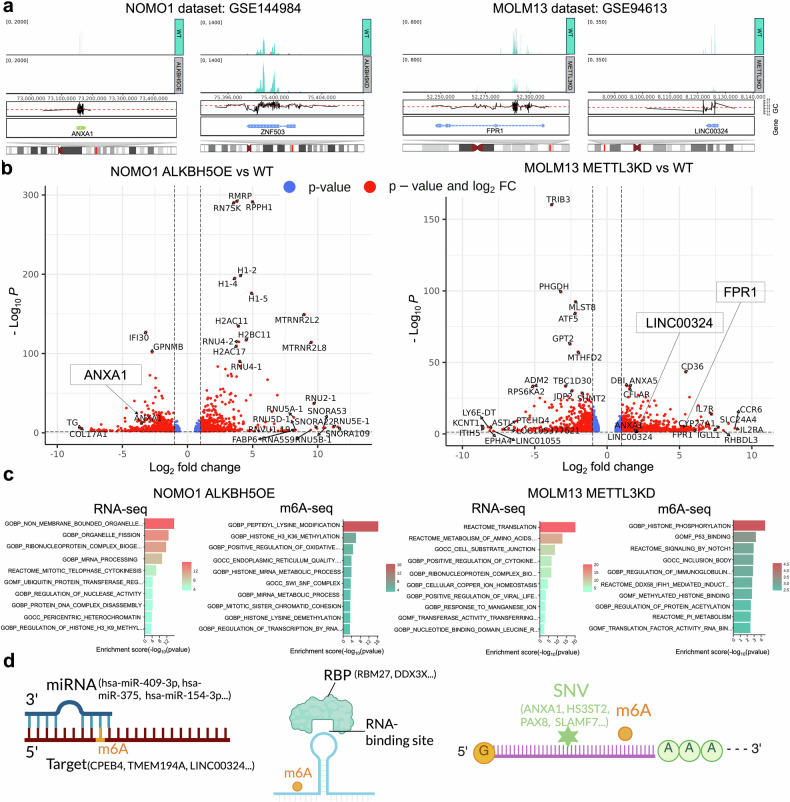
Differential RNA expression and modification analysis and functional annotation in two AML cell lines (left, NOMO1; right, MOLM13). **a** Differential m6A peak visualization (blue, m6A methylation; pink, RNA input/control). **b** Volcano plots for DEG visualization. The symbol names of the top-ranked genes of interest are labeled. **c** Bar plots for GO functional annotation. **d** Interactome of RNA modifications (left, miRNA target; middle, RBP; right, SNV). The highlighted genes were selected by intersecting significantly methylated genes with known or predicted binding sites of RBPs and miRNAs from RMBase

To understand the underlying RNA modification-mediated mechanisms underlying the biological functions of RMPs, we sought to identify potential downstream targets of RMPs. First, by analyzing bulk transcriptome and epitranscriptome data from human cell lines subjected to various manipulations of RMPs (e.g., METTL3 knockdown), we identified 51,180 significant DEGs and 482,054 peaks with significant gain/loss of RMs, respectively (Supplementary Tables 1 and 2). Then, we described the differential modification peaks between conditions (e.g., METTL3 knockdown vs. wild-type) with annotations of gene names and types, revealing 33,698 transcripts with peak changes (Supplementary Table 2). We focused on METTL3- and ALKBH5-overexpressing (OE) or knockdown (KD) AML models, in which these RMP targets overlapped with previously identified malignant transition-associated genes^13^ (e.g., ANXA1 and FPR1). Specifically, ALKBH5-OE significantly reduced both ANXA1 mRNA m6A methylation and expression in AML cells, whereas an increase in m6A and a decrease in ZNF503 expression were detected in the ALKBH5-KD AML cell line (NOMO1, Fig. 3a, b). Next, functional annotation analyses were performed for DEGs and differential peaks/transcripts via signatures from MSigDB, including Gene Ontology, hallmark, cell type and reactome. Our analysis of differentially expressed or methylated genes in ALKBH5-OE NOMO1 cells revealed enrichment of GO terms such as mRNA processing and histone H3 K9 methylation (Fig. 3c, Supplementary Table 3) or histone H3 K36 methylation, histone mRNA metabolic process and miRNA metabolic process (Fig. 3c, Supplementary Table 4). Similarly, FPR1 transcripts whose m6A level significantly decreased and whose expression significantly increased were found in the METTL3-KD AML cell line (MOLM13, Fig. 3a, b). DEGs in METTL3-KD MOLM13 cells compared with control cells were enriched in terms related to eukaryotic translation initiation, metabolism of amino acids and derivatives, positive regulation of cytokine production, ribonucleoprotein complex biogenesis and neutrophil degranulation (Fig. 3c, Supplementary Table 3). Additionally, m6A-methylated transcripts upon METTL3 KD in MOLM13 cells were involved in histone phosphorylation, p53 binding, signaling by NOTCH1, regulation of immunoglobulin production, protein serine threonine tyrosine kinase activity and mononuclear cell differentiation (Fig. 3c, Supplementary Table 4). These analyses suggested that the deposition of RMs modulated RNA processing and affected the translation of key target mRNAs, with downstream effects on protein synthesis and cell function.

We subsequently investigated the colocalization of RMs with RBPs and miRNA targets and the subcellular localization of modified transcripts. For each dataset, we inferred potential posttranscriptional regulatory interactions via RMBase v3.0,^2^ identifying the RNA modification sites that were located within the annotated RBP binding regions or miRNA target sites. For example, in METTL3-KD MOLM13 cells, we observed reduced m6A levels at miRNA target sites in genes such as CPEB4, TMEM194A, and LINC00324 (targeted by miRNAs, including hsa-miR-409-3p, hsa-miR-375, and hsa-miR-154-3p; Fig. 3d and Supplementary Table 5). These findings suggest that the overlap of miRNA interaction regions with m6A sites may influence METTL3 binding to RNAs. More interestingly, LINC00324 expression was upregulated in METTL3-KD MOLM13 cells compared with control cells (Fig. 3a, b), and LINC00324 has been previously reported to suppress tumor growth in vivo.^14^ This example suggests a potential interplay between miRNAs and m⁶A modification, which may influence the structure, function or stability of downstream targets and cellular processes; however, further experimental confirmation is needed.

While not directly assessing RNA subcellular localization changes, we noted that both METTL3 and LINC00324 are localized in the nucleus and cytoplasm according to existing data (Supplementary Table 6), and prior RNA-FISH studies supported the dual localization of LINC00324.^14^ Moreover, RBPs such as RBM27 and DDX3X overlapped with differential m6A sites on ANXA1 in ALKBH5-OE NOMO1 cells (Fig. 3d and Supplementary Table 7), suggesting a possible interaction relationship that warrants further experimental validation. In addition, genetic mutations in genes such as ANXA1, HS3ST2, PAX8, and SLAMF7 may occur at m6A methylation sites, potentially contributing to a significant reduction in the m6A levels of downstream target genes, as observed in ALKBH5-OE NOMO1 cells (Fig. 3d and Supplementary Table 8).

### RNA modification regulatory patterns at the cell type level across the human single-cell transcriptome

**Fig. 4:**
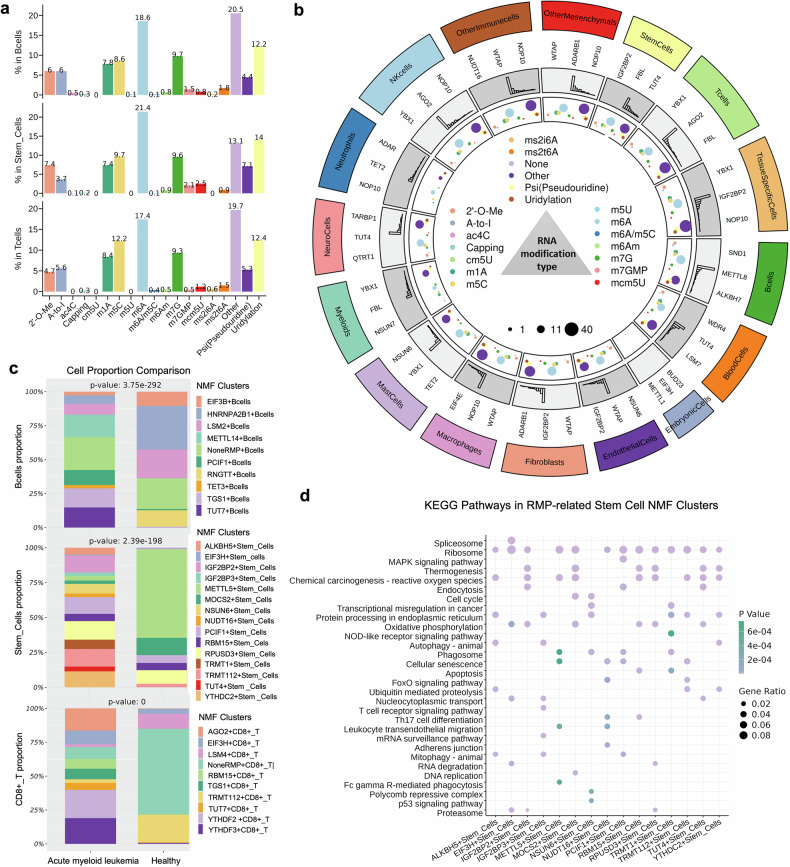
Regulatory patterns of RNA modifications at the cell type level. **a** Bar plots showing the percentage of RNA modification-related NMF clusters in three representative major cell types. **b** Circos plot depicting the statistics of RMP-mediated NMF clusters for 16 major cell type classes (first layer). The second layer represents the top 3 RMPs annotated for NMF clusters of each major cell class. Tissues belonging to the same organ were placed under the same cluster and marked with the same color. In the fourth layer, the color and size of a circle represent the RM type and enzyme count, respectively. **c** Bar plot revealing the proportions of RNA modification-specific NMF clusters with significant enrichment of RMP marker genes (denoted as RMP+ cell types) in the AML and control groups. **d** Dot plot showing the top 10 activated KEGG pathways in RNA medication-related stem cell clusters according to the DEGs among these groups (*p* < 0.05)

In total, 264 scRNA-seq datasets spanning 62 human tissues were used to investigate the expression profiles and biological roles of regulatory enzymes, including those that catalyze, remove and recognize RNA modifications (Supplementary Table 9). A total of 7538 RNA modification special NMF clusters were identified via the expression of 179 RMPs (Supplementary Table 9) and the top-ranked DEGs among these subclusters (Supplementary Table 10, see “Methods”). Compared with RNA modifications in 16 major cell type classes (Supplementary Fig. 2), m6A regulatory enzymes presented widespread expression across most cell types, suggesting broad potential involvement in cell type-specific RNA regulation (Fig. 4a, Supplementary Fig. 3). Specifically, m6A exhibited the highest proportion of related modification signatures among tissue-specific clusters (e.g., parenchymal and epithelial cells), stem cells, endothelial cells, fibroblasts, macrophages, mast cells, neutrophils, NK cells, and other innate immune or mesenchymal cells (Fig. 4a, Supplementary Fig. 3). In addition, m7G- and m5C-related regulatory enzymes were most frequently enriched in embryonic and myeloid cell clusters, respectively, suggesting cell type-specific regulatory associations (Fig. 4b). T and B cells are also commonly affected by other modification types (e.g., m3C through METTL8; Figs. 4a, b). After the global gene expression levels of RMPs across cell types were assessed, NOP10, YBX1, NSUN6, IGF2BP2, WTAP, and AGO2 were frequently annotated for most cell types (Fig. 4b), indicating their wide involvement in physiological processes via the deposition of pseudouridine, m5c, m6A and m7G methylation.

We further compared the proportions of RNA modification-associated NMF clusters across disease states. Our analysis of the GSE185381 dataset, an AML scRNA-seq dataset, revealed that the proportions of METTL14 + B cells, ALKBH5+ stem cells and AGO2+ CD8+ T cells were significantly greater in AML patients than in healthy individuals (Fig. 4c). To better decipher the regulatory patterns of RNA modifications in heterogeneous cell populations across various human diseases, we performed differential expression analysis to obtain the markers for each NMF cluster. For example, a total of 3,376 genes were differentially expressed in the ALKBH5+ stem cells of GSE185381. KEGG enrichment analysis of the DEGs revealed that the cluster of ALKBH5+ stem cells presented activated functions related to protein processing in the endoplasmic reticulum, chemical carcinogenesis (reactive oxygen species), autophagy (animal), ubiquitin-mediated proteolysis, ribosome, and mitophagy (animal) (Fig. 4d). Notably, over 55.2% of the marker genes overlapped with the genes mentioned above as differentially expressed or differentially methylated in AML cells following ALKBH5 overexpression or knockdown (GSE144984, Supplementary Fig. 3), highlighting a substantial set of shared downstream targets of ALKBH5 regulation. Intriguingly, among the ALKBH5 target genes identified in the NOMO cell line, ZNF503 and ANXA1 were differentially expressed in the ALKBH5+ stem cell subcluster (Supplementary Table 11). Moreover, ANXA1 was identified as a significant DEG in pseudobulk comparisons of AML stem cells versus their healthy counterparts (Supplementary Table 12). The functional role of ANXA1 has been well established in AML^15^ and other cancers.^16^ ZNF503 has only been implicated in tumor progression in solid malignancies,^17–19^ including hepatocellular carcinoma, which demonstrated that elevated mRNA and protein expression of ZNF503 promotes tumor progression and initiation,^19^ indicating its potential relevance in AML biology. In parallel, cellChat analysis revealed key ligand‒receptor pairs with high interaction strength, such as MIF--CD74 + CXCR4, MIF--CD74 + CD44, MDK + NCL, LGALS9 + CD44, and ITGB2 + ICAM2, facilitating communication from ALKBH5+ stem cells to tumor microenvironment cells (e.g., T and B cells, Supplementary Table 13). Notably, many of those ligands and receptors were not only upregulated in pseudobulk AML cells but also identified as ALKBH5 targets across multiple cell lines (such as NOMO1, THP1, and HeLa; Supplementary Tables 1 and 2). This example implies that the modification of stem cells with m6A methylation through ALKBH5 may enhance RNA modification-mediated interactions, promoting AML progression.

To experimentally validate our interesting observations, we examined the ALKBH5 protein level via intracellular flow cytometry. CD34^+^ immature AML blast cells (representing leukemia stem/initiating cell [LSC/LIC] populations) were enriched in the primary AML patient sample (Supplementary Fig. 4a). ALKBH5 levels were significantly higher in AML patients than in healthy controls (Supplementary Fig. 4a) and were particularly enriched in LSCs/LICs compared with CD34⁻ AML bulk cells (Supplementary Fig. 4a), suggesting its potential role in sustaining the self-renewal of AML stem cells. To investigate the function of ALKBH5 in LSC/LIC leukemogenesis, we first overexpressed ALKBH5 in AML cells (Supplementary Fig. 4b). The upregulation of ALKBH5 significantly promoted cell proliferation, the cell cycle and DNA replication (Supplementary Fig. 4b). Moreover, ALKBH5 overexpression increased the mRNA levels of ZNF503 in HL60 cells (Supplementary Fig. 4c), and ALKBH5 knockdown resulted in the downregulation of ZNF503 in HEK-293T cells (Supplementary Fig. 4d), which is consistent with in silico predictions. To determine the role of ALKBH5 in LSC/LIC self-renewal, we sorted CD34+ cells from AML cells (representing the LSC/LIC population) and overexpressed ALKBH5 in this cell population. The results revealed that ALKBH5 upregulation significantly increased cell proliferation, the cell cycle and apoptotic resistance (Supplementary Fig. 5a). Conversely, knockdown of ALKBH5 significantly impaired LSC/LIC self-renewal (Supplementary Fig. 5b). However, further in vivo lineage tracing or serial transplantation assays will be needed to conclusively determine whether ALKBH5 directly drives leukemia stem cell self-renewal.

Collectively, the results of integrative computational and experimental analyses implied that ALKBH5 functions as an important m6A demethylase in AML, regulating critical target transcripts at the posttranscriptional level; affecting the mRNA processing, stability and translation of protein processing-related genes; and further driving leukemogenesis and stem cell self-renewal.^11^

### Cell type-specific somatic variants associated with RNA modification and human diseases

**Fig. 5:**
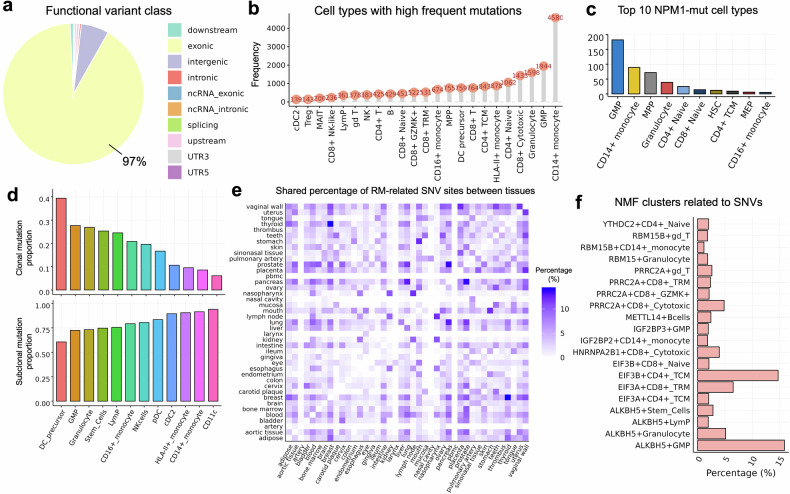
Cell-type somatic variants associated with RNA modification. **a** Pie plots showing the percentage of functional variants from different classes. **b** The frequency of the top 20 somatic mutations across different cell types detected in an AML dataset. **c** Bar plot revealing the frequency of the top 10 cell types carrying NPM1 mutants. **d** Bar plots showing comparisons of clonal and subclonal mutation proportions among the innate immune cells of AMLs. **e** Heatmap demonstrating the percentage of shared RNA modification-related SNV sites between each of the 41 tissue types. **f** Bar plot showing the percentage of the top 20 RNA modification NMF clusters related to SNVs in GSE185381

Over 10 million single-cell somatic variants were identified in over 1 million cells from 1227 diseased samples of 120 scRNA-seq datasets. These include 8,103,997 (~93%) exonic single nucleotide variants (SNVs) and ~ 7% other types (such as intergenic), with significant functional impact prioritized on the basis of our criteria (see “Methods”) at the single-cell level (Fig. 5a). To explore the roles of genes of interest in cellular processes and diseases from a variation perspective, we included variant information for mutated cells across specific tissues and datasets on the single-cell transcriptomics page of the RMzyme website. For example, in the AML dataset GSE185381, 490 mutated genes were detected (Supplementary Table 14). Among the top 20 frequently mutated genes (e.g., GSTP1, MNDA, TPT1, and NPM1), a significant enrichment was observed in innate immune cells (e.g., CD14+ monocytes, granulocytes and GMPs) and stem cells (e.g., MPPs) (Fig. 5b). Notably, NPM1, the most commonly mutated gene in adult AML,^20^ presented SNVs in the largest number of HSC, MPP, GMP and CD14+ monocytes of AMLs (Fig. 5c). All variants occurring in NPM1 were missense mutations (p.E105K) located in the exonic region with high pathogenic likelihood (fathmm scores = 0.91, Supplementary Table 14). These observations suggest that early driver mutations, such as NPM1, occur in HSCs, which, owing to their self-renewing nature, might generate clones that evolve into preleukemic clones and multipotent progenitor leukemia stem cells, initiating tumorigenesis.^21^ We next examined the clonality patterns at cell-type resolution by analyzing the distributions of the clonal and subclonal SNVs. Among innate immune cells, GMP and DC precursor cells generally presented a greater clonal proportion, whereas granulocytes and CD14+ monocytes, CD11c+ DCs and cDCs presented greater subclonal proportions (Fig. 5d).

To better understand how somatic mutations affect RNA modifications in diseased samples and their potential pathogenic or carcinogenic mechanisms, we linked the cell-level variants to RNA modification sites. Initially, on the basis of the aforementioned mutations, these high-confidence variants were further mapped to known SNV-related RNA modification sites^2^ by leveraging genomic coordinates and identifying the nearest RNA modification positions. Then, SNVs within 10 bases of an RNA modification site were classified as potentially linked to RNA modification processes. To identify reliable RNA modification-related variants at the cell type level, those SNVs with consistency between the RM types defined in RMBase V3.0^2^ and the scRNA-seq-derived NMF clusters annotated via RMPs were ultimately retained. This resulted in 9650 SNVs located in close proximity to RNA modification sites in 92,246 cells across 41 tissue types (Supplementary Table 15). Annotation data, including associated diseases, modification types, and gene symbols, enriched the functional insights of the identified SNVs related to RNA modification from both bulk and single-cell samples and were integrated and provided in the single-cell transcriptome module. By focusing on the tissue specificity of these variants, we further calculated the percentage of RNA modification-related SNV sites shared between any pair of tissues. Intriguingly, we found that the most significant tissue pairs included breast-thyroid, lung-pancreatic, prostate-pancreatic, uterus-vaginal wall, and stomach-mouth (Fig. 5e). We speculated that tissues sharing either developmental origins or other forms of physiological linkage may also exhibit molecular similarities in epitranscriptomic patterns.^22–24^ In particular, tissues harboring shared SNVs near RNA modification sites might be prone to similar pathological processes. For example, both breast and thyroid tissues are known to be hormone sensitive and are frequently affected by cancers with overlapping regulatory features.^25,26^ As a representative example in our analysis, the NOP14 p.I779V (chr4:2938298--2938299) variant was detected in ADAR+ tissue-specific cells from breast cancer and in ADARB1+ stem cells from thyroid cancer (Table 3). The ADAR protein family comprises key regulatory enzymes mediating A-to-I editing, a process implicated in disease progression, including in breast and thyroid malignancies.^27–29^ In addition, DNA mutations of NOP14 I779V were reported in COSMIC,^30^ and NOP14 RNA was also reported to harbor adenosine sites that can be edited by ADAR1, whose expression level is linked with the regulation of cancer pathogenesis.^31–33^ A representative illustration of potential RNA modification-linked variants demonstrated the utility of the RMzyme to identify driver-like editing events in carcinogenesis at single-cell resolution. We believe that these correlative observations will guide future rigorous validation in cancer cells that may share vulnerabilities or regulatory networks across tissue types with similar mutational or RNA modification profiles.

**Table 3:** Representative tissue-specific A-to-I editing single-cell variants collected in RMzyme

barcode	GCTCCTAAGTCCGGTC--brca9--Mature_luminal_cells--GSE174588	CAACGATTCAAGAGGC--ATC13--Stem_like_cells--GSE193581	TGTAGACGTTCCACGG--ATC13--Stem_like_cells--GSE193581
**scSNV id**	chr4:2938298-2938299: -		
**Hugo Symbol**	NOP14		
**Variant Classification**	Missense Mutation		
**tx**	NM_001291979		
**exon**	exon17		
**txChange**	c.A2335G		
**aaChange**	p.I779V		
**Variant Type**	SNP		
**Func.refGene**	exonic		
**tissue**	breast	thyroid	
**disease**	precancerous lesion from brca1 mutation carriers	anaplastic thyroid cancer	
**NMF cluster**	ADAR+Tissue_Specific_Cells	ADARB1+Stem_Cells	
**Modification type**	A-to-I		

Additionally, by focusing on 15,113 SNV sites in GSE185381, we found that ALKBH5 is the most frequently associated RMP, with 4044 SNV occurrences in AMLs (Table 4). Other m6A-related proteins, such as METTL14 (366), IGF2BP3 (531), and YTHDC2 (530), also presented no Supplementary Table NV associations but presented lower frequencies. This highlights the prominence of the m6A-related modification machinery in SNV-related events in AMLs. NMF clusters associated with ALKBH5, such as ALKBH5 + GMP (2394), ALKBH5+ granulocyte (772), and ALKBH5+ stem cells (423), are also the most common (Fig. 5f). These clusters represent specific cell types where ALKBH5-associated SNVs are enriched. The frequent association of SNVs with ALKBH5 suggests that these variants may influence m6A RNA demethylation, potentially altering posttranscriptional regulation in specific cell types (e.g., innate immune or progenitor cells). This phenomenon is particularly notable in hematopoietic progenitor cells such as GMPs and stem cells, where dynamic RNA modifications are critical for cell fate decisions.^11,34^ Given the role of ALKBH5 in removing m6A marks, the enrichment of SNVs in ALKBH5+ cells may imply that disruption of m6A demethylation^35^ leads to aberrant mRNA metabolism (e.g., stability, translation).^36,37^ In general, our analysis provides a novel framework for uncovering the interplay between RNA modifications and genetic variations in disease contexts and at the cell type level.

**Table 4:** Statistics for single cells in GSE185381 carrying somatic variants related to RMPs

RMP	Cell count	RNA modification type
ADAR	2	A-to-I
ALKBH5	4044	m6A
ALYREF	183	m5C
EIF3A	1413	m6A
EIF3B	2589	m6A
EIF3H	332	m6A
FMR1	8	m5C
FXR1	161	m6A
HNRNPA2B1	1012	m6A
HNRNPC	30	m6A
IGF2BP2	539	m6A
IGF2BP3	531	m6A
LRPPRC	12	m6A
METTL14	366	m6A
METTL5	106	m6A
MOCS2	16	Other
PRRC2A	1789	m6A
RBM15	448	m6A
RBM15B	518	m6A
RBMX	158	m6A
SAFB	47	m6A
SAFB2	12	m6A
SND1	26	m6A
WTAP	28	m6A
YBX1	12	m5C
YTHDC2	530	m6A
ZC3H13	201	m6A

### Protein expression profiles, regulation of posttranslational modifications and mutation maps of RNA modification enzymes

**Fig. 6:**
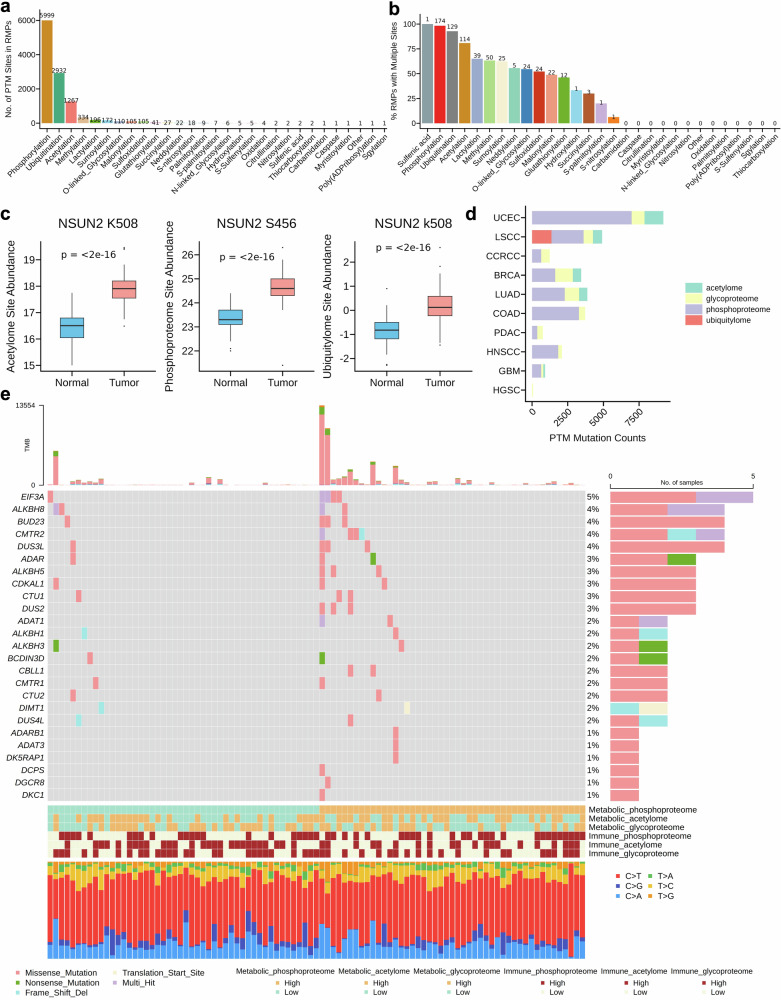
Proteogenomic information on RNA-modifying enzymes. **a** Number of PTM sites per RMP type. **b** Proportion of modified RMPs with multiple PTM sites. **c** Differential expression of NSUN2 at acetylation, phosphorylation, and ubiquitination sites in CPTAC tumor samples vs. normal samples. **d** Number of RMP mutations near PTM sites by CPTAC cancer type. **e** Somatic mutations in RMPs of UCEC samples and their immune-metabolic features

We first collected and collated publicly available PTM sites from related databases and conducted an extensive literature search (Supplementary Tables 16, 17).^38–45^ Our manual curation of PTM information revealed 28 well-defined PTM types and 11,378 PTM sites in 179 human RNA-modifying enzymes (Fig. 6a). The PTM sites were widespread, with each RMP containing one or more PTM sites (Supplementary Table 16). As expected, phosphorylation was the most common PTM type, followed by ubiquitylation, acetylation, methylation and lactylation (Fig. 6a). Notably, ~96% of RMPs had more than one PTM type; for example, 17 PTM types were identified in HNRNPA2B1 (a regulator of m6A methylation; Supplementary Table 16). Most RMPs have multiple PTM sites (Fig. 6b), highlighting the complexity and dynamic nature of PTMs, which may impact the functions of RMPs.

Using CPTAC proteogenomic data, we aimed to better understand the PTM sites that regulate protein‒protein interactions and the fundamental molecular events that contribute to multiple cancers. We analyzed the expression profiles of mRNA transcripts, total protein abundance, and site-specific modifications for four types of PTMs—acacetylation, phosphorylation, glycosylation, and ubiquitylation—across 11 cancer types. A total of 1371, 1353, 5700, 264, and 1688 potential interacting enzymes that regulate the activity of RMPs and control PTM deposition or removal were identified at the RNA, protein, phospho-site, acetyl-site, and ubiquitination-site levels, respectively, on the basis of their significant correlations (|correlation coefficient| ≥ 0.7 and *p* < 0.01). Our results also revealed that 80 RMPs were aberrantly expressed in 11 types of tumors compared with normal samples (log2FC ≥ 1 and *p* < 0.05). For example, NSUN2, a m5C methylation writer, consistently upregulated acetyl- and phospho-site and ubiquitination-site levels in tumors (Fig. 6c). Additionally, among its predicted interactor proteins, RPA1, NCL, AARS, LIN7C, RDX, UBA1, and UBE3A were identified to physically interact with NSUN2 with experimental evidence (Supplementary Table 17).

The transient and reversible nature of PTMs enables a quick response to adapt to changes in the microenvironment under immune and metabolic responses.^46^ We first characterized the activity of metabolic and immune-related pathways in cancers by applying gene set variation analysis (GSVA) from the “GSVA” package^47^ to PTM data. We then sought to better understand the potential regulation of PTM sites on proteins, considering that the mutation would also alter protein functions. The mutation maps of 179 RMPs were constructed via CPTAC somatic mutation data as well as information on PTM regulation of immunometabolism. Interestingly, higher mutation frequencies in RMPs, such as EIF3A, CMTR2, and ALKBH5, were observed in cancers with greater metabolic activity (Fig. 6e). This pattern implies that potential disruption of RNA modification control impacts metabolic pathways. For example, ALKBH5 mutations can reduce the demethylation of glycolytic gene transcripts, promoting their stability and translation; indeed, ALKBH5 is known to facilitate glycolysis by stabilizing GLUT1/4, HK2, LDHA, and G6PD in multiple cancer contexts.^48,49^ Similarly, mutations in EIF3A may dysregulate metabolic programs by affecting AMPK signaling and mitochondrial anabolism.^50,51^

We also intersected our PTM information with mutation data, identifying a total of 30,245 cancer mutations at or within 10 residues of PTM sites (Supplementary Table 18). Among the cancer types analyzed, uterine corpus endometrial carcinoma (UCEC) is the most mutation-rich, which is consistent with its known characteristics of mismatch repair deficiency and genomic instability. Among them, 337 mutations that coincided with or around the position of 4 categorized PTM-modified sites were identified in 89 RMPs (Supplementary Table 18). For example, a PTM-linked frameshift mutation (p.N97Mfs*13) in YTHDC1 was observed in UCEC, around which lysine (K90) was identified to be acetylated in UCEC patients (Supplementary Table 18). This finding indicated that acetylation upstream of loss-of-function mutations could stabilize the truncated YTHDC1 protein, which persistently loses its capacity to recognize or bind to m6A, resulting in aberrant splicing of mRNA and tumorigenesis.

## Discussion

Posttranscriptional modifications alter the canonical ribose and base structure to determine RNA fates, further affecting various biological processes and cellular phenotypes. Defects in enzyme regulation and changes in RNA modification patterns are closely related to disease occurrence and progression.^3^ In addition, various PTMs may modify protein structure, activity and function, highlighting the therapeutic potential of targeting PTMs on RMPs. Hence, dissecting the dynamics and functional roles of RMPs from a multiomics perspective constitutes the very first step toward establishing therapeutic strategies for various human diseases. In this study, we present a comprehensive characterization of the RNA modification landscape across epitranscriptomes of human cell lines under different experimental conditions, single-cell transcriptomes and proteogenomes of human tissues, advancing our understanding of the roles of RNA modifications in regulating cellular responses and human diseases. Through the integration of bulk and single-cell transcriptomics with epitranscriptomics and (PTM) proteomics, we identified distinct regulatory patterns and molecular mechanisms underlying RNA modification-mediated gene expression, RNA stability and translation.

First, our analyses reveal an intricate map of RMs, with over 2 million sites identified across eight modification types in various cell lines and experimental conditions. Among these, m6A has emerged as the most prevalent modification, with its distribution spanning diverse RNA regions, including CDSs, UTRs and ncRNAs. Interestingly, while m1A, m5C, m7G, and pseudouridine showed localized enrichment patterns, the widespread distribution of m6A underscores its centrality in transcriptome regulation. These observations align with previous reports suggesting a major role of m6A in RNA metabolism and cellular processes that require broad genomic coverage.^52^ These distinct positional preferences likely reflect the unique biogenesis and recognition mechanisms of each modification type, suggesting that functional specificity is tailored to diverse cellular needs. However, it is important to note that the greater number of m6A peaks observed in our dataset likely reflects both biological prevalence and methodological sensitivity. Current m6A profiling techniques (e.g., MeRIP-seq and m6A-CLIP/m6A-seq) are mature and widely adopted, whereas other modifications, such as ac4C or m1A, often suffer from limited detection sensitivity and fewer public datasets.^53^ Therefore, our data summary likely represents a combination of true biological abundance and research bias toward better-studied modifications rather than an absolute measure of modification prevalence. Next, differential modification analysis highlighted the role of RMPs in shaping transcriptomic responses under various conditions of perturbation experiments. For example, METTL3 KD and ALKBH5 OE/KD in AML cells resulted in substantial changes in RNA modification patterns and downstream gene expression. Notably, key transcripts, such as FPR1, LINC00324, ANXA1, and ZNF503, presented coordinated shifts in m6A deposition and expression levels, supporting the hypothesis that m6A influences RNA stability and translation in a context-specific manner. Functional enrichment analyses further linked these changes to critical biological pathways, including translation initiation, immune regulation, and tumorigenesis, suggesting that RNA modifications act as fine-tuners of cellular responses. The m6A-forming enzyme METTL3 and eraser ALKBH5 have been linked to monocyte inflammatory responses and oncogenesis in AML,^54^ with ANXA1 potentially playing a role in stem cell dysfunction and tumorigenesis by promoting monocyte differentiation toward M1 macrophages and inflammation.^55^ Moreover, potential interactions among miRNA target sites (as exemplified by LINC00324), RBPs such as RBM27 and DDX3X,^56^ which influence RNA translation in LSCs, and mutations in genes such as ANXA1 with RNA modifications mediated by METTL3 and ALKBH5 were identified in AMLs. These examples underscore factors and cofactors implicated in m6A regulation, such as impacting the m6A methylation level or contributing to m6A deposition or blocking demethylation. They also suggest that our database provides useful information on the multifaceted regulatory networks mediated by RMPs.

Expanding these insights to the single-cell level, we demonstrated that RNA modification patterns exhibit remarkable cell-type specificity, with m6A dominating in epithelial, stem, and immune cells. The enrichment of m6A-related machinery components, such as WTAP and ALKBH5, in specific cell populations suggests their pivotal roles in modulating cell fate decisions and immune responses. In the context of AML, our identification of ALKBH5+ stem cells with unique RNA modifications and ligand‒receptor interaction profiles provide mechanistic insights into leukemogenesis and tumor microenvironment interactions. These findings are consistent with prior reports on the role of m6A in regulating stem cell self-renewal and differentiation while highlighting its involvement in disease progression. Overall, our observations from enrichment analyses (KEGG and RMP expression-based clustering) provide valuable clues for functional modulation and will guide downstream experimental studies. Our subsequent analysis of somatic variants associated with RNA modifications further bridges the gap between cell-type genetic alterations and epitranscriptomic regulation in human diseases. By integrating somatic variant data with RNA modification maps, we revealed novel insights into how genetic variations potentially influence RNA modification landscapes at single-cell resolution. For example, the high prevalence of missense mutations in NPM1 within hematopoietic stem and progenitor cells further supports the hypothesis that early driver mutations can impact the RNA modification machinery, thereby altering cellular differentiation and proliferation pathways. The frequent association of ALKBH5 with somatic variants in AML suggests a potential link between disrupted m6A demethylation and leukemogenesis. The enrichment of ALKBH5-associated variants in progenitor cells, such as GMPs and hematopoietic stem cells, supports a model in which dysregulated m6A dynamics contribute to early clonal expansion and malignant transformation.^11,36,37^ Given the relatively low somatic mutation burden of AML compared with that of solid tumors, germline variants may significantly contribute to its pathogenesis. Several AML studies have reported that pathogenic variants, often in GATA2, RUNX1, and CEBPA, lead to familial predispositions to AML, which are characterized by dramatic immunodeficiency related to profound B lymphocyte, NK, and dendritic cell deficiencies.^57^ These findings highlight the need to investigate the functional consequences of both somatic and inherited mutations on RNA modifications in a cell type-specific manner.

Finally, our analysis of PTMs in RNA-modifying proteins highlights an additional layer of regulation influencing RMP function. The widespread occurrence of phosphorylation, acetylation, and ubiquitination sites in RMPs underscores their dynamic and context-dependent regulation. For example, the upregulation of NSUN2 and its PTM-related interactors in tumors suggests the coordinated regulation of RNA methylation and protein interaction networks in cancer. Notably, the intersection of the PTM and mutation data revealed cancer-specific mutations that potentially altered RMP activity and RNA modification. For example, a frameshift mutation in YTHDC1 associated with acetylation in UCEC illustrates how PTM-mutation crosstalk may stabilize truncated proteins, impair RNA modification recognition, and promote tumorigenesis. Lactylation of the zinc-finger domain of METTL3 enhances its binding affinity for m6A-modified Jak1 mRNA together with YTHDF1, activating STAT3 to promote downstream pro-oncogene expression in colon cancer-infiltrating myeloid cells.^58^ Phosphorylation inhibits ALKBH5 demethylase activity, increasing the mRNA m6A levels of DNA damage repair genes and increasing their mRNA stability and expression. K48-linked polyubiquitination of ALKBH5 induces its degradation, thereby decreasing its protein level without altering its demethylating activity. High expression of the deubiquitinase USP9X in AML^37^ results in increased ALKBH5 expression and AML cell proliferation. PTMs of ALKBH5, such as ubiquitination and SUMOylation, promote AML cell survival^37^ and play roles in the maintenance of oncogenesis.^59^ Hence, PTMs are an integral part of tumor cell adaptation and response to intracellular and environmental changes. A deeper understanding of PTM-related processes leading to cancer initiation and progression has the potential to reveal novel therapeutic targets, identify biomarkers of the response to existing therapies, and increase our knowledge of cancer therapeutics.

Overall, this study highlights the complex interplay among RNA modifications, genetic variations, and PTM regulation, advancing our understanding of RNA modification-mediated biology in health and disease. The cell type-specific and context-dependent regulatory patterns identified here provide a foundation for future investigations into the functional implications of RMs and their potential as therapeutic targets. RMzyme is the first unique database that uses hundreds of multiomics datasets to comprehensively characterize mechanisms across a wide range of RNA modification types and regulatory enzymes. We believe that RMzymes provide valuable resources for gaining profound insights into the intricate regulation of RMPs at the molecular and cellular levels. Moreover, it serves as a pivotal tool for identifying targetable biomarkers, facilitating the development of enhanced therapeutics.

## Materials and methods

### Ethics statement

This paper describes studies involving human tissue samples that were approved by the Ethics Committee of West China Hospital of Sichuan University (Approval Number: 2024-2067). All tissue samples were collected and used in compliance with informed consent policies. Written informed consent was obtained from the human participants. All studies were performed in accordance with the Declaration of Helsinki.

### Systematic collection of multiomics datasets

We collected hundreds of multiomics datasets from 7 public databases, encompassing genomics, bulk and single-cell transcriptomics, epitranscriptomics, proteomics and posttranslational proteomics across 63 human tissue types (Supplementary Table 19). To detect high-confidence RNA modification sites and call condition-specific peaks through reprocessing of the raw sequencing data, the epitranscriptomic datasets were selected on the basis of the following stringent criteria: (1) Availability of raw sequencing data (e.g., fastq, bam or bigwig files) and metadata from original articles or relevant GEO datasets. (2) Clear documentation of experimental conditions (e.g., input vs. IP samples, biological replicates). (3) Inclusion of proper controls (e.g., negative controls or wild types, knockdowns of RNA-modifying proteins). (4) Sufficient sequencing depth and quality metrics (e.g., base quality, mapping rate). Among them, RNA modification data focused on profiling human cell lines subjected to various manipulations of RMPs of 8 different modification types (m1A, ac4C, m5C, m5U, m6A, m6Am, m7G, and pseudouridine) were curated from the NCBI GEO database. These datasets apply different RNA modification profiling techniques, including NGS techniques at the base-resolution level, such as miCLIP, and techniques with limited resolution, such as m5C-RIP-seq, bisulfite-seq, MeRIP-seq, m6A-seq, and m7G-RIP-seq (Supplementary Table 20). The processed count matrix data and raw sequencing data of the scRNA-seq datasets across the 6 platforms (i.e., 10X Genomics, Microwell-seq, InDrop V2, MARS-seq, BD Rhapsody and Seq-well) were obtained from repositories such as ArrayExpress, NCBI GEO, NCBI SRA, HTAN, GSA and dbGAP. The processed data of the CPTAC proteogenomic datasets were downloaded from the Proteomic Data Commons (PDC). Additionally, we downloaded the experimentally identified modifications and gathered a collection of 179 RNA-modifying enzymes in humans from over 100 studies and public databases, including RMDisease,^60^ RMBase,^2^ m7GHub,^7^ m6A-Atlas,^5^ m5C-Atlas^6^ and NCBI PubMed.^1,61,62^

### Identification and annotation of RMs

The raw RNA modification sequencing data from the cell lines were processed via the following procedures. (1) Read processing: Raw sequencing data quality was assessed via FastQC (version 0.11.5). The raw reads were subjected to adapter removal via Cutadapt (version 4.8) and trimmed via Trimmomatic (version 0.39)^63^ with the default settings. Cleaned reads were then aligned to the human genome assembly hg19 (ENSEMBL, version 74) or hg38 (ENSEMBL, version 102) according to their original HISAT2 (version 2.2.1).^64^ To minimize the rate of false positives, only uniquely mapped reads with −*q* ≥ 20 selected via SAMtools (version 1.6)^65^ were retained for the subsequent analysis of each sample. In addition, for the inputs of RIP-seq or MeRIP-seq, the expression levels of genes (gene counts) were quantified via featureCounts (version 1.6.4),^66^ a tool that converts aligned short reads into read counts for each sample. (2) Peak detection and comparison: Peaks enriched in immunoprecipitated samples over corresponding input samples were called via MACS2 (version 2.2.7.1)^67^ with the parameters “--nomodel --extsize 200 -f BAM -q 0.01”. (3) Peak change detection between conditions and peak annotation: For differential RNA modification analysis, overlapping peaks identified in both biological replicates from each condition (i.e., with and without treatment) were merged via the mergePeaks command in the homer (version 5.0.1).^68^ The consensus peaks were then annotated on the basis of Ensembl (version 74 or 102) gene annotation information via intersectBed from BEDTools.^69^ Reads aligned to peaks were also counted via featureCounts. The peak counts were normalized via the DESeq2 negative binomial distribution model. Differences in RNA modification patterns and gene expression between conditions were determined via the R packages DESeq2 (version 1.42.0) and edgeR (version 4.0.16). Enrichment analysis was performed for the DEGs as well as the genes with significantly increased/decreased modification peaks via the enricher() function of the clusterProfiler R package (version 4.10.0).^70^ The metagene profiles were plotted via the Guitar R package (version 2.18.0). Visualization of genome coverage and peak identification was performed with the ggcoverage R package (version 1.4.0)^71^ on the basis of bigWig or bed files. The relationships between RNA modifications and miRNA targets, RBP binding events and SNV events were analyzed through overlapping with differential RNA modification sites for each dataset.

### Processing of single-cell RNA sequencing data

After obtaining the scRNA-seq datasets from previously published studies (Supplementary Table 19), we applied CellRanger (version 7.0.1) and dropest (version 0.8.6) to preprocess the datasets with the raw reads (bam, sra and fastq files). For the count matrix data, we excluded cells with low-quality transcriptomes following similar filtering steps. Specifically, cells with <200 and >7000 detected genes and cells with more than 10% mitochondrial reads were removed. We further filtered out samples with fewer than 30 remaining cells before conducting downstream analysis. We also retrieved and manually curated the corresponding metadata and cell annotation data from PCTanno,^13^ the DISCO database^72^ and original publications to ensure harmonization across datasets and facilitate downstream data integration. To analyze the landscape of RNA-modifying enzymes in human tissues, we first integrated the diseased samples with their accompanying healthy controls from the same tissue type within each disease dataset. We profiled all cells that were annotated with 582 minor cell types at fine granularity, in which we categorized them into 16 major cell types (Supplementary Table 9). The preliminarily integrated gene expression and phenotype matrix for the scRNA-seq data was then parsed with the Seurat R package (version 5.1.0)^73^ to create Seurat objects for individual major cell types. The gene expression profile for each cell subtype was subsequently analyzed via the Seurat::SCTransform() function^74^ on the basis of the top 2000 variable genes. Uniform manifold approximation and projection (UMAP) was used to visualize the cell subtypes.

### Nonnegative matrix factorization of RNA modification regulators in cell subpopulations

By using the NMF R package (version 0.21.0),^75^ the NMF algorithm was performed separately on each cell type annotated for each dataset. All these steps were performed as described in previous studies.^76,77^ Specifically, NMF was applied to each SCTransform result for different rank values, and then, we extracted consensus modules for each optimal NMF rank that was identified for each sample. We utilized NMF programs in each sample to characterize the effect of RNA modification-mediated regulator expression on cell subpopulations, each summarized by its top-scoring genes.

### Marker gene identification and functional enrichment analysis for NMF RNA modification-related cell types

Seurat::FindAllMarkers() was utilized to find markers for each NMF cluster of each cell subtype, with the parameters min.pct, logfc.threshold and max.cells.per.ident being set as 0.2, 0.25 and 250, respectively. On the basis of the DEGs among these NMF cell clusters, the marker genes were screened out on the basis of two criteria: (a) the highest log2-fold change difference (absolute value of avg_log2FC) for each NMF cluster and (b) intersection with 179 RNA-modifying proteins (Supplementary Table 9). The RNA modification-related NMF clusters (denoted as RMP+ cell subtypes) were defined as subclusters where RMPs were significantly overrepresented among the top marker genes. The detailed annotations for these clusters enriched for RNA modification regulators are listed in Supplementary Table 10 and were used for conducting downstream analysis.

The markers differentially expressed among NMF-derived RNA modification-related subclusters were further identified for each cell subtype. We primarily used signatures from the Kyoto Encyclopedia of Genes and Genomes (KEGG) database through clusterProfiler to detect gene sets that were significantly enriched (with adjusted *p* < 0.05) for these NMF clusters.

### Cell‒cell interaction (CCI) analysis of NMF RNA modification-related clusters

We inferred cell‒cell interactions and constructed communication networks among our identified NMF cell clusters of diseased human tissues via CellChatDB in CellChat (version 2).^78^ The netVisual_circle() function was then used to show the strength or weakness of the CCI networks from the target cell cluster to different cell clusters for all NMF RNA modification-related clusters. NetVisual_bubble() shows bubble plots of significant ligand‒receptor interactions between the target cell cluster and other NMF subclusters.

### Single-cell somatic mutation detection

SComatic is a tool that provides functionalities to detect somatic single-nucleotide mutations in high-throughput single-cell genomics and transcriptomics datasets, such as single-cell RNA-seq. We run SComatic to detect somatic mutations in scRNA-seq data with aligned sequencing reads in BAM format for all epithelial cells. The input BAM file contains the cell type barcode information in the cell barcode tag “CB” generated from cellranger or other drop-seq tools. We also applied a fathmm filter^79^ to all the cells. On the basis of this machine learning approach, each mutant of a single cell was assigned a score about the likelihood of a given SNV/INDEL being pathogenic. Only variants computationally predicted to be pathogenic (Fathmm score > 0.7) and with a variant allele frequency (VAF) > 0.05 were retained for further analysis. We subdivided mutations into clonal and subclonal fractions by setting a CCF threshold of 0.9^80^ to examine the clonality patterns during pathophysiological changes at cell-type resolution across different tissues. The mutational load for single cells was estimated as the number of somatic mutations per haploid genome normalized by the breadth of coverage.^81^ By leveraging RNA modification sites that interact with SNVs reported in RMBase^2^ and the genomic coordinates of SComatic variants, SNV loci with the nearest RNA modification positions (within 10 bases of an RNA modification site) were identified on the basis of the previous computational framework.^82^

### Proteogenomic data analysis

We performed differential expression and correlation analyses via CPTAC proteogenomic data. The detailed procedures are as follows. (1) For transcriptomic data (RNA), differential expression was implemented via Limma-Voom from the limma R package (version 3.58.1). The trimmed mean of *M* values (TMM) between-sample normalization was applied to counts via calcNormFactors, the voom transformation was applied via limma::voom(), and limma::lmFit() was used for the moderated *t* test, followed by empirical Bayes shrinkage with limma::eBayes(). FDRs were computed via the Benjamini‒Hochberg procedure. (2) For proteomic and posttranslational proteomic data, we first used K-nearest neighbors (KNNs) to impute missing values with impute.knn() from the impute R package (version 1.76.0) within each dataset. Differential expression was subsequently performed via Limma on median-MAD normalization matrices output from SpectrumMill version 7.08. limma::lmFit() was subsequently used for the moderated *t* test, followed by empirical Bayes shrinkage with limma::eBayes(). No imputation was performed prior to differential expression analyses. Proteins and PTM sites were filtered out if they were present in <10 patients in either group being compared. (3) Spearman’s rank correlation coefficient was used to evaluate the associations of 179 RNA modification enzymes with the expression levels of RNA and protein, as well as the relative abundance of all phosphoproteome, acetylome, glycoproteome and ubiquitylome sites.

### Experimental method details

#### Primary patient and healthy donor samples

Human primary AML and healthy donor samples were collected after written informed consent was obtained. Mononuclear cells (MNCs) were isolated via Ficoll‒Paque density gradient centrifugation (10335693, Cytiva). The cells were cultured in SFEM (09650, STEMCELL) supplemented with 20% fetal bovine serum (FBS), 1% penicillin–streptomycin, and the following recombinant human cytokines: stem cell factor (rhSCF, 100 ng/mL, 250--03, PeproTech), thrombopoietin (rhTPO, 10 ng/mL, 300--18, PeproTech), Flt-3 ligand (rhFlt-3 L, 10 ng/mL, 300--19, PeproTech), interleukin-3 (rhIL-3, 10 ng/mL, 200--03, PeproTech), and interleukin-6 (rhIL-6, 10 ng/mL, 200--06, PeproTech).

#### Lentiviral infection

For HL60 leukemia cells, transduction with pLV3-CMV-ALKBH5-3×FLAG-CopGFP-puro viral supernatant was performed as follows: six-well plates were coated with 10 μg/mL recombinant fibronectin fragment (retronectin, T110A, Takara) overnight at 4 °C. HL60 cells were seeded onto coated wells and incubated with viral supernatant, followed by centrifugation at 1000 × *g* for 2 h at room temperature. GFP-positive cells were subsequently sorted via flow cytometry and expanded in culture.

#### Cell surface and intracellular staining

Human primary AML cells were washed with ice-cold PBS and stained with an anti-CD34-647 antibody (5 μL/test, CL647-98145-4, Proteintech) for 1 h at 4 °C. After washing, the cells were fixed in 4% paraformaldehyde (BL539A, Biosharp) at 4 °C for 20 min with rotation. Fixed cells were permeabilized in 0.3% Triton X-100 (1139ML100, BioFROXX) for 10 min at room temperature, resuspended in PBS, stained with a rabbit anti-human ALKBH5 antibody (1:200, 16847-1-AP, Proteintech) for 1 h at 4 °C, and mixed every 10 min. After washing, the cells were incubated with an Alexa Fluor 488–conjugated goat anti-rabbit IgG (H + L) secondary antibody (GRAR003, Proteintech) for 30 min at room temperature. The stained cells were washed twice with PBS and analyzed by flow cytometry.

#### Cell cycle and proliferation assays

The cell cycle status was evaluated via a Cell Cycle Detection Kit (KGA9101-50, KeyGEN BioTECH) with PI staining according to the manufacturer’s protocol. Cell proliferation was assessed via an EdU-647 Cell Proliferation Detection Kit (C0081S, Beyotime) following the manufacturer’s instructions. Flow cytometry was performed, and the data were analyzed with FlowJo software.

#### RNA extraction and quantitative real-time PCR

Total RNA was extracted via an RNA extraction kit (RE-03113, Foregene). cDNA synthesis was carried out with HiScript III All-in-One RT SuperMix (R333-01, Vazyme) using 1000 ng of total RNA. Quantitative PCR was performed via the use of SupRealQ Ultra Hunter SYBR qPCR Master Mix (Q713-00, Vazyme) on a QuantStudio Real-Time PCR System (Applied Biosystems). The sequences of primers used were as follows:

**Table:** 

Gene	Primer
ZNF503-F	TCAAGCCGTACTCCAAACCC
ZNF503-R	CCGACTTCTCCGACGAAACAC
ALKBH5-F	CGGCGAAGGCTACACTTACG
ALKBH5-R	CCACCAGCTTTTGGATCACCA

#### Colony formation assay

CD34⁺ cells were sorted from HL60 cells stably overexpressing ALKBH5 via flow cytometry. Sorted cells were plated at a density of 5 × 10³ cells in methylcellulose medium supplemented with 2 mg/mL puromycin (H4434; StemCell Technologies). After incubation at 37 °C for 10 days, colonies were counted.

For ALKBH5 knockdown, CD34⁺ cells were sorted from HL60 cells by flow cytometry and transfected with ALKBH5 siRNA via an RNA transfection reagent (E607402, Sangon Biotech). At 24 h post-transfection, the cells were plated at 5 × 10³ cells per well in methylcellulose medium (H4434; StemCell Technologies) and incubated at 37 °C for 10 days before colony counting. At 48 h post-transfection, the cells were harvested for cell cycle and apoptosis assays.

#### Cell cycle analysis

The cell cycle distribution of the ALKBH5-overexpressing and ALKBH5-knockdown cells was assessed via a commercial Cell Cycle Detection Kit (KGA9101-50; KeyGENE BioTECH) and analyzed via flow cytometry.

#### Apoptosis analysis

Apoptosis in ALKBH5-overexpressing and ALKBH5-knockdown cells was evaluated via an apoptosis detection kit (CB105-20T, Oriscience) according to the manufacturer’s instructions. The samples were analyzed via flow cytometry.

### Statistical analysis

The standard tests employed in the present study included Student’s *t* test, the Wilcoxon rank-sum test, the Kruskal‒Wallis test, and the chi‒square test. These tests were utilized to assess variations in continuous target or categorical variables across different subgroups for comparison. The descriptions of the statistical details and methods are provided in the figure legends, text, or methods. The experimental data were analyzed via GraphPad Prism 8 and are presented as the means ± SDs. *P* values were computed with a two-sided and unpaired Wilcoxon rank-sum test. Routine statistical analyses of this study were performed in R v4.3.3, and a two-sided *p* value less than 0.05 was deemed statistically significant.

## Supplementary information

Supplementary Meterials

Supplementary Tables

## Data Availability

All datasets analyzed in this study were published previously and are publicly available. This work relied on the curation and integrative analysis of external studies. Thus, the curated datasets are available at the download page of our website (https://rh-luo.cn/RMzyme), including accession numbers for the primary datasets and the results of multiple downstream analyses. Additional datasets from unpublished studies will be added when possible. The pipeline used for data analysis is described on the statistics page of our website. The related scripts for analyzing the data reported in this paper are available on GitHub (https://github.com/RH-LUO/RMzyme).
