# Supplementary material for: RMzyme: regulations of RNA-modifying enzymes in humans
Source: Signal Transduct Target Ther. 2026 Feb 12;11:52. doi: 10.1038/s41392-025-02568-2 (PMC12895048; doi:10.1038/s41392-025-02568-2)

Supplementary Materials for

RMzyme: regulations of RNA-modifying enzymes in humans

Ruihan Luo^1,2,3#^, Haixia Xu^1,2,3#^, Qingbo Zhou^1#^, Shanli Ding^4^, Min Qiang^5^, Jianguo Wen^2^, Pora Kim^2^, Xiaojuan Yang^1^, Yunshi Cai^1,6,7^, Kunlin Xie^1,6,7^, Jiang Zhu^1,6^, Yungang Xu^5*^, Tian Lan^1,6,7*^, Xiaobo Zhou^2,8*^, Hong Wu^1,6,7*^

Correspondence to: wuhong@scu.edu.cn, xiaobo.zhou@uth.tmc.edu, blue_sky_land@163.com and yungang.xu@xjtu.edu.cn

**This PDF file includes:**

Figures. S1 to S6

Captions for Tables S1 to S20

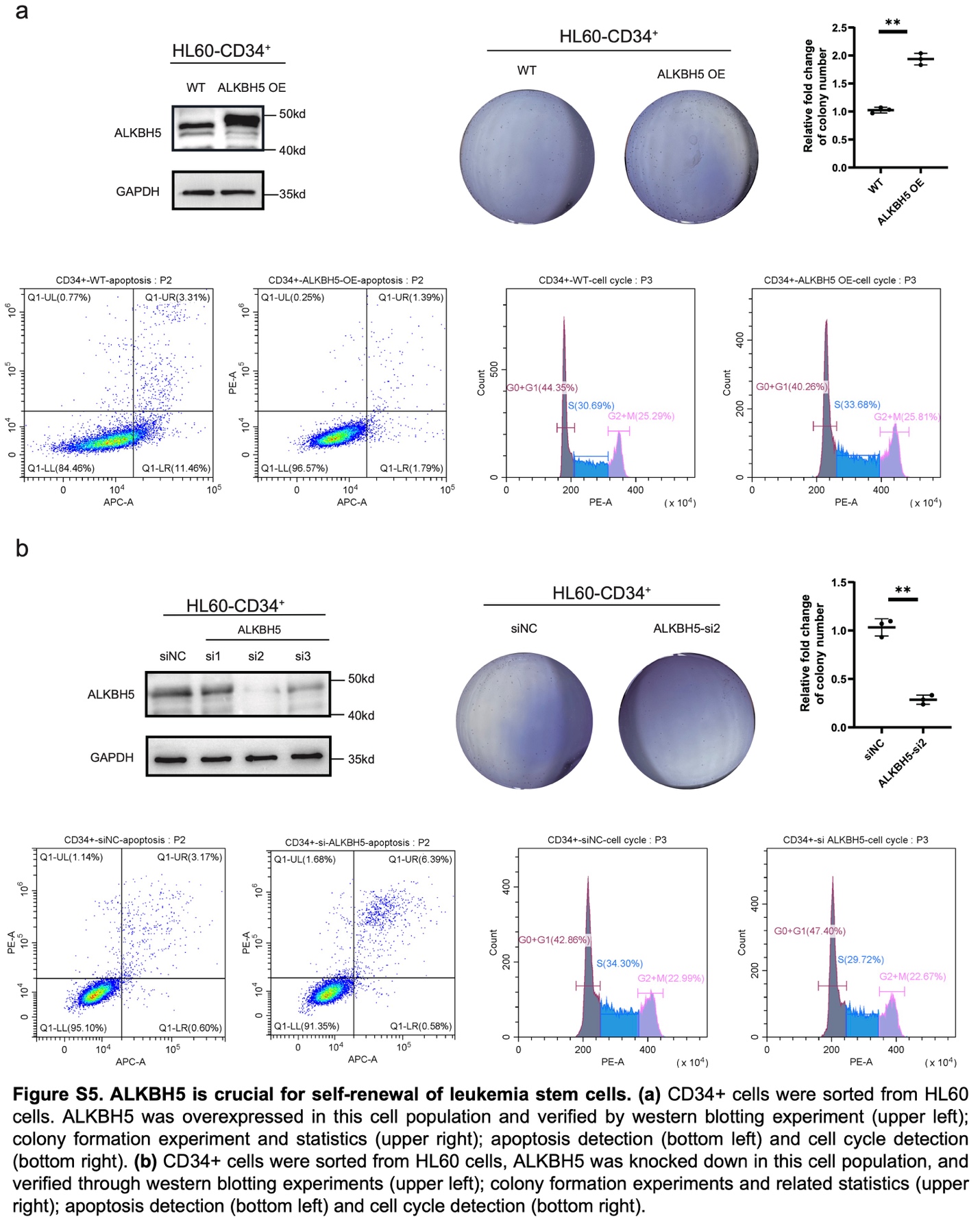

Supplement: Supplementary file 1 — Supplementary Meterials [file 41392_2025_2568_MOESM1_ESM.docx]
